# Influence of an Antioxidant Nanomaterial on Oral Tablet Formulation: Flow Properties and Critical Quality Attributes

**DOI:** 10.3390/antiox14070829

**Published:** 2025-07-05

**Authors:** Andrea C. Ortiz, Javiera Carrasco-Rojas, Sofía Peñaloza, Mario J. Simirgiotis, Lorena Rubio-Quiroz, Diego Ruiz, Carlos F. Lagos, Javier Morales, Francisco Arriagada

**Affiliations:** 1 Escuela de Química y Farmacia, Facultad de Ciencias, Universidad San Sebastián, Santiago 7510157, Chile; andrea.ortizo@uss.cl; 2 Departamento de Ciencias y Tecnología Farmacéutica, Facultad de Ciencias Químicas y Farmacéuticas, Universidad de Chile, Santiago 8380494, Chile; 3 Instituto de Farmacia, Facultad de Ciencias, Universidad Austral de Chile, Campus Isla Teja, Valdivia 5090000, Chile; 4 Chemical Biology & Drug Discovery Lab, Escuela de Química y Farmacia, Facultad de Ciencias, Universidad San Sebastián, Campus Los Leones, Lota 2465 Providencia, Santiago 7510157, Chile; 5 Centro Basal Ciencia & Vida, Fundación Ciencia & Vida, Av. del Valle Norte 725, Huechuraba 8580702, Chile

**Keywords:** antioxidant nanomaterial, nanoantioxidant, mesoporous silica nanoparticles, caffeic acid, powder flow properties, antioxidant tablets

## Abstract

Antioxidant nanomaterials, particularly mesoporous silica nanoparticles (MSNs) functionalized with polyphenols, offer innovative solutions for protecting oxidation-sensitive components and enhancing bioavailability in pharmaceuticals or extending the shelf life of nutraceutical and food products. This study investigates the influence of MSNs functionalized with caffeic acid (MSN-CAF) on powder flow properties and their tableting performance. Aminated MSNs were synthesized via co-condensation and conjugated with caffeic acid using EDC/NHS chemistry. Antioxidant capacity was evaluated using DPPH^●^, ABTS^●+^, ORAC, and FRAP assays. Powder blends with varying MSN-CAF concentrations (10–70%) were characterized for flow properties (angle of repose, Hausner ratio, Carr’s index), tablets were produced via direct compression, and critical quality attributes (weight uniformity, hardness, friability, disintegration, nanoparticle release) were assessed. MSN-CAF exhibited reduced antioxidant capacity compared with free caffeic acid due to pore entrapment but retained significant activity. Formulation F1 (10% MSN-CAF) showed excellent flowability (angle of repose: 12°, Hausner ratio: 1.16, Carr’s index: 14%), enabling robust tablet production with rapid disintegration, low friability, and complete nanoparticle release in 10 min. Additionally, the antioxidant nanomaterial demonstrated biocompatibility with the HepG2 cell line. MSN-CAF is a versatile nanoexcipient for direct compression tablets, offering potential as an active packaging agent and delivery system in the nutraceutical and food industries.

## 1. Introduction

Surface-functionalized nanoparticles with antioxidants, also known as nanoantioxidants, have emerged as a new generation of antioxidant agents [1,2]. These systems overcome significant limitations of natural antioxidants, which are prone to activity loss during processing or storage because of their sensitivity to pH, light, oxygen, and heat [3]. Additionally, they can enhance the transport and dissolution rate of poorly water-soluble polyphenols, thereby improving bioavailability [4,5]. Consequently, antioxidant nanomaterials have been investigated for applications in biomedicine, the pharmaceutical industry, cosmetics, the food industry, and nutraceuticals [6,7,8,9,10]. In particular, silica-based antioxidant nanomaterials have garnered significant interest due to the unique properties of the silica matrix. Silicon dioxide nanoparticles possess a highly versatile surface that enables the incorporation of functional groups, facilitating covalent bonding or physical adsorption of various polyphenols. These surface advantages, combined with the versatility of the silica matrix, allow for the modification of particles to achieve porosity, a hollow cavity to encapsulate bioactive molecules, or degradability in response to specific stimuli. Furthermore, colloidal silicon dioxide is classified as a Generally Recognized as Safe (GRAS) material and is used as a food additive (E-551). However, mesoporous silica nanoparticles (MSNs) as drug delivery carriers have not been commercialized in the pharmaceutical industry, though they have been extensively studied [11,12]. Notably, their incorporation into oral tablets has been reported. Ibrahim A. H. and colleagues utilized a 3^2^ full factorial design to optimize a formulation containing silymarin-loaded MSNs for the production of lyophilized tablets [13]. Similarly, Kim D. et al. reported tablets containing celecoxib-loaded MSNs to enhance drug loading and subsequent dissolution [14]. Additionally, Abbaraju P. et al. used MCM-41 particles to fabricate floating tablets [15]. However, despite reports of MSN incorporation into tablets, critical technological parameters for their formulation remain underexplored. Powder flow properties are influenced by (i) steric repulsions, (ii) friction forces, and (iii) cohesive forces [16]. On one hand, steric repulsion and cohesive forces are associated with grain geometry and interactions such as liquid bridges, electrostatic charges, magnetic dipole–dipole interactions, or van der Waals forces, respectively. On the other hand, friction forces depend on both the surface state (rough or smooth) and the chemical nature of the powders [17]. The predominance of one of these forces over the others results from the microenvironmental conditions and the physicochemical properties of the grains. When nanoparticles are blended with different excipient powders, the nanoparticles are expected to adsorb onto the surface of the powder particles, with either homogeneous or heterogeneous dispersion. In this context, the distribution is primarily influenced by the surface properties of the nanoparticles. The hydrophobic or hydrophilic nature of antioxidants in nanomaterials can influence their interparticle interactions, which in turn affect the final rheological properties of the powder. However, limited studies are available on the influence of mesoporous silica nanoparticles on powder flow properties, and even fewer address antioxidant nanomaterials. To the best of our knowledge, there are no reports on the incorporation of silica-based nanoantioxidants into oral tablets. Because of their properties, MSN-based antioxidant nanomaterials can serve as nanoexcipients to protect oxidation-sensitive components in formulations, nanocarriers to enhance the apparent solubility and transport of bioactive molecules across membranes, and agents with inherent antioxidant activity. This is of significant importance in the pharmaceutical, cosmetic, and nutraceutical industries, adding value to formulations and extending product shelf life by acting as an active packaging agent.

Here, we investigated the influence of a silica-based antioxidant nanomaterial on the rheological properties of powders blends used to produce oral tablets by direct compression. To this end, we synthesized, using a cocondensation method, aminated porous silica nanoparticles onto which caffeic acid was immobilized. Caffeic acid is a naturally occurring polyphenol found in a wide variety of plants, including coffee, fruits, and vegetables [18]. It is one of the most potent antioxidants within the hydroxycinnamic acid family [19] and the predominant member of that family in the human diet [20], being linked to various pharmacological benefits [21,22,23]. In addition to the above, which highlights its importance and interest for conjugation as an antioxidant molecule, it offers several operational advantages. Among these, its relatively hydrophilic nature stands out, facilitating its incorporation into aqueous solutions; it possesses structural simplicity compared with other antioxidant molecules, such as flavonoids; and it features a carboxylic group, which enables its direct conjugation to various surfaces [24,25,26,27]. Although the antioxidant activity of caffeic acid is known to rely on both its catechol group and acrylic acid moiety [28], the literature suggests that the contribution of the carboxylic acid is moderate compared with that of the catechol [29,30]. Therefore, conjugation via this functional group may ensure that its activity is not drastically reduced. These characteristics make caffeic acid an attractive molecule for easy incorporation onto the surface of nanomaterials, which is why it was selected. This antioxidant nanomaterial was thoroughly characterized, and its antioxidant capacity was evaluated using DPPH^●^, ABTS^●+^, ORAC, and FRAP assays. The influence of this nanomaterial on the flow properties of a powder for direct compression was assessed by varying the proportion of the antioxidant nanomaterial and other excipients. From a formulation with optimal properties, oral tablets containing the antioxidant nanomaterial were manufactured, and their critical quality attributes were evaluated.

## 2. Materials and Methods

Tetraethyl orthosilicate (TEOS, 98%), and (3-aminopropyl)triethoxysilane (APTES, ≥98%), caffeic acid (CAF, ≥98%), ethyl-3-(3-dimethylaminopropyl)carbodiimide) (EDC, ≥98%), n-hydroxysuccinimide (NHS, 98%), ammonium hydroxide solution (NH_4_OH, 28–30%, ACS reagent), Folin–Ciocâlteu reagent, 2,2-diphenyl-1-picrylhydrazyl (DPPH^●^), 2,2′-azinobis-(3-ethylbenzothiazoline-6-sulfonic acid (ABTS), fluorescein, 2,2′-Azobis(2-methylpropionamidine) dihydrochloride (AAPH, 97%), and 2,4,6-tripyridyl-s-triazine (TPTZ, ≥98%) were purchased from Merck (Merck KGaA, Darmstadt, Germany). Cetyltrimethylammonium bromide (CTAB, 99%), hydrochloric acid fuming (HCl, 37%, ACS grade), and absolute ethanol (EtOH, ACS grade) were obtained from PanReac AppliChem ITW Reagents (Darmstadt, Germany). All reagents were used without prior purification. All experiments were carried out using deionized water (Milli-Q, 18.2 MΩ·cm).

### 2.1. Antioxidant Nanomaterial Synthesis

#### 2.1.1. Synthesis of Aminofunctionalized Mesoporous Silica Nanoparticles (MSN-NH_2_)

For the synthesis of MSN-NH_2_, a previously reported co-condensation method was modified [31]. In a round-bottom flask, 0.82 g of CTAB was dissolved in 560 mL of water at 40 °C. After the temperature reached 25 °C, the mixture was stirred for 45 min. Then, a mixture of APTES (1.8 mL) and TEOS (6.9 mL) was added dropwise to the aqueous solution of CTAB. Subsequently, 4.5 mL of NH_4_OH (25%) was added and allowed to react for 4 h at room temperature with constant stirring. The MSN-NH_2_ was collected by centrifugation and washed thoroughly. The surfactant was removed by solvent extraction, leaving the nanoparticles suspended in an acidic ethanol solution (0.6 M HCl) under constant stirring and reflux for 6 h. Finally, three washes with water and one with ethanol were performed. This process was performed twice.

#### 2.1.2. Functionalization of MSN-NH_2_ with Caffeic Acid (MSN-CAF)

The MSN-NH_2_ was surface functionalized with caffeic acid following a previously reported method [32]. Briefly, in a round-bottom flask CAF (11.1 mM), EDC (22.2 mM), and NHS (44.4 mM) were dissolved in water, using ethanol as cosolvent. The mixture was stirred for 10 min, after which an aqueous nanoparticle suspension (10 mg/mL) was added. The reaction mixture was then allowed to proceed for 6 h at room temperature. The nanoparticles conjugated with CAF (MSN-CAF), i.e., antioxidant nanomaterials, were collected by centrifugation and washed. They were then dried at 60 °C under vacuum for 12 h and collected as powder.

### 2.2. Preparation of Tablet Formulation

The tablet formulation (125 mg) consisted of MicroceLac^®^ 100 (75% alpha-lactose monohydrate and 25% microcrystalline cellulose), crospovidone, magnesium stearate, and the antioxidant nanomaterial (or MSN-NH_2_ as control). These materials were selected because they are among the most commonly used excipients in tablet compression processes, ensuring robustness and reproducibility in formulation development [33]. Briefly, MicroceLac^®^ 100 was selected as a coprocessed excipient that combines lactose and microcrystalline cellulose, offering both good compressibility and improved flow properties, which are essential for direct compression [34]. Crospovidone was included as a superdisintegrant to promote rapid tablet disintegration [35], and magnesium stearate was selected as a lubricant to prevent sticking and ensure good flow during tableting [36,37]. Given that crospovidone is typically used at 2–5% in tablet formulations, with changes in its proportion causing significant variations in disintegration time [38,39], we maintained its concentration as constant to isolate and evaluate the effects specifically associated with varying nanoparticle proportions. Additionally, to maintain appropriate tablet weight and ensure adequate lubrication for proper compression, the proportions of MicroceLac^®^ 100 and magnesium stearate were adjusted accordingly.

The impact of the antioxidant nanomaterial on the angle of repose, flowability, and compressibility properties was assessed by varying the ratio of MSN-CAF (or MSN-NH_2_) to MicroceLac in four different formulations (Table 1). All powders in the formulation were mixed manually [40].

### 2.3. Materials Characterization

The hydrodynamic diameter and zeta potential of the nanoantioxidant were measurement in triplicate using a Zetasizer Nano ZS90 (Malvern, London, UK), with a detection angle of 173° and an equilibration time of 120 s. Textural properties such as specific surface and pore volume of the nanomaterials were calculated by the Brunauer–Emmett–Teller (BET) and Barrett–Joyner–Halenda (BJH) methods, using a N_2_ adsorption/desorption isotherm measured at −196 °C on a Micromeritics 3-Flex instrument (Micromeritics Instruments, Norcross, GA, USA). FT-IR was used to analyze the vibrational modes of the characteristic functional groups of MSN-NH_2_ and MSN-CAF. Infrared spectra were obtained using a Nicolet iS5 FT-IR spectrometer (Nicolet Instrument, Thermo Fisher Scientific, Waltham, MA, USA) in the range 650 to 4000 cm^−1^, with a resolution of 4 cm^−1^ and 16 scans per spectrum to generate an average. To analyze the shape and surface of samples (nanoantioxidant, powder, and tablet), scanning electron microscopy (SEM) images were taken on an FEI inspect F50 model microscope (FEI, Hillsboro, OR, USA) with an accelerating voltage of 10.00 kV. Thermogravimetric analysis (TGA) was conducted using a Netzsch TG 209 F1 (Netzsch-Gerätebau GmbH, Selb, Germany) with an airflow of 20 mL/min and a heating rate of 10 °C/min, over a temperature range of 30–800 °C.

Nanoantioxidant samples were suspended in ethanol. An aliquot was taken and deposited onto a silicon grid, dried, and then coated with gold. The powder blend or tablet samples were deposited directly on the grid and then coated with gold.

### 2.4. Characterization of Antioxidant Activity and Total Phenolic Content

#### 2.4.1. Determination of Total Phenolic Content

The total phenolic content (TPC) was determined using the Folin–Ciocâlteu method [41]. Each sample was dissolved (or suspended in the case of nanoparticle samples) in distilled water and mixed with 10% Folin–Ciocâlteu reagent, followed by incubation for 2 min at 40 °C. Subsequently, 5% Na_2_CO_3_ was added, and the mixture was incubated for an additional 20 min at 40 °C. The absorbance of the samples was measured at 765 nm using a microplate reader (BioTek Instruments, Inc., Winooski, VT, USA). The results are expressed as milligrams of gallic acid equivalents per gram of powder (mg GAE/g powder).

#### 2.4.2. DPPH Radical Scavenging Assay

The concentration required to scavenge 50% of the DPPH radical (SC_50_) was determined. Briefly, 50 μL of the sample (free CAF or antioxidant nanomaterial) was mixed with 150 μL of a DPPH^●^ solution in ethanol (156 μM) and incubated for 30 min in the dark. The absorbance was then measured at 517 nm using a microplate reader. The results were expressed as μg/mL [42].

#### 2.4.3. ABTS^●+^ Scavenging Assay

The concentration required to scavenge 50% of the ABTS radical (SC_50_) was determined. Briefly, 50 μL of each sample was mixed with 150 μL of a 169 μM ABTS radical solution and incubated for 30 min in the dark. The absorbance was measured at 732 nm using a microplate reader. Similarly as for the DPPH^●^ assay, the results were expressed as μg/mL [43].

#### 2.4.4. Oxygen Radical Absorbance Capacity (ORAC) Assay

The ORAC assay was performed using a microplate reader as previously described [42]. Briefly, 45 μL of the sample and 175 μL of a fluorescein solution (108 nM) were pre-incubated for 30 min at 37 °C. Then, 50 μL of AAPH (18 mM) was added to the mixture, and the fluorescence was measured every 2 min for 2 h at excitation and emission wavelengths of 480 nm and 520 nm, respectively. The results are expressed as micromoles of Trolox per gram of powder (μmol Trolox/g powder).

#### 2.4.5. Ferric-Reducing Antioxidant Power (FRAP) Assay

The FRAP assay, based on the reduction of Fe^3+^-TPTZ to Fe^2+^-TPTZ, was performed using a microplate reader following established procedures [43]. Briefly, 10 μL of the sample was mixed with 290 μL of FRAP reagent, and the mixture was incubated for 60 min in the dark. The absorbance was then measured at 593 nm. The results were expressed as micromoles of Trolox per gram of powder (μmol Trolox/g powder).

### 2.5. Rheological Properties

#### 2.5.1. Angle of Repose

The static angle of repose (θ) was determined according to USP specifications using a funnel positioned 4 cm from the top of the cone-like pile of powder sample. After the powder flowed through the funnel, the angle of repose for each sample was calculated using Equation (1). The test was conducted in triplicate (n = 3).

(1) $$\tan\theta =\frac{Height\;(cm)}{Radius\;(cm)}$$

#### 2.5.2. Powder Flowability

The flowability of powder mixtures was evaluated using the Hausner ratio (Equation (2)) and the compressibility index (Carr’s index, Equation (3)). Measurements of bulk and tapped volumes were performed with an ETD-1020 tap density tester (Electrolab, Mumbai, India). Powder mixtures were placed in a graduated cylinder, and the untapped bulk volume (V_0_, mL) was recorded. After 500 taps, the final tapped volume (V_f_, mL) was measured. The test was conducted in triplicate (n = 3).

(2) $$Hausner\;ratio=\frac{V_{0}}{V_{f}}$$

(3) $$Compressibility\;Index=\frac{V_{0}-V_{f}}{V_{0}}\times 100$$

The flow properties were classified according to the criteria outlined in USP <1174> (2024) [44], based on the angle of repose, Hausner ratio, and Carr’s index values, as presented in Table 2.

### 2.6. Tablets Manufacturing

The tablets were manufactured by direct compression of the previously described powder blend. The process was carried out using D-type punches with a diameter of 5 mm. The compression speed was set to level 3, and the compression hardness to level 5, using a D-8 Piccola Classica rotary tablet press (Riva, Buenos Aires, Argentina). In each compression batch, the first 10 tablets were discarded.

### 2.7. Characterization of the Critical Quality Attributes of the Final Product

#### 2.7.1. Weight Uniformity and Tablet Dimensions

Weight uniformity was assessed in accordance with the specifications outlined in the monograph for each finished product. As no active pharmaceutical ingredient was used in this study, no specific weight uniformity requirements were stipulated. However, based on the punch type used during manufacturing, a target tablet weight of 120 mg (with an acceptable range of 90–110%) was optimized. After tablet production, 10 tablets were randomly selected and individually weighed using an analytical balance. Tablet dimensions were measured using a vernier caliper. The diameter was determined by positioning the caliper jaws around the tablet’s circumference, while the thickness was measured between the flat surfaces.

#### 2.7.2. Tablet Hardness

Tablet hardness was evaluated using a durometer (Erweka, Langen, Germany). The assessment was conducted on each production batch, with 10 tablets randomly selected for testing.

#### 2.7.3. Friability Test

Friability was evaluated according to USP (2024). For tablets with a unit mass of 650 mg or less, a sample of whole tablets equivalent to approximately 6.5 g was used. For tablets with a unit mass greater than 650 mg, 10 whole tablets were selected. Before testing, the tablets were carefully dedusted, weighed, and placed in the drum of a friability tester. The test was conducted by rotating the drum 100 times. After testing, the tablets were removed, dedusted, and accurately weighed. Friability was calculated using Equation (4), with an acceptance criterion of weight loss of less than 1%.

(4) $$Friability\;\left(\%\right)=\frac{Initial\;weight-final\;weight}{Inicial\;weight}\times 100$$

#### 2.7.4. Disintegration Time

Disintegration was assessed in 900 mL of phosphate buffer (pH 6.8) at 37.0 ± 0.5 °C using six tablets. The disintegration time was recorded for each tablet. The test was performed using a two-bowl disintegrator with six tablet compartments (Erweka, Germany).

#### 2.7.5. Nanoparticle Release Test

An in vitro release study was adapted to investigate the release behavior of nanoparticles from an oral tablet using a stirring technique. The release medium consisted of 10 mL of buffer solutions at pH 1.2, 4.5, and 6.8, maintained at 37 °C, based on reported procedures [45,46,47]. The buffer compositions are shown in Table 3.

At specific time points (2, 5, 10, 15, 20, 30, 45, and 60 min), 1 mL aliquots of the release medium were withdrawn and replaced with an equal volume of fresh medium. The collected samples were sonicated, filtered, and then centrifuged at 13,000 rpm for 20 min. The resulting nanoparticle pellet was dried and weighed to determine the cumulative release of nanoparticles at each time point.

### 2.8. Assessment of Cellular Metabolic Activity

The human hepatocellular carcinoma line HepG2 (ATCC HB-806) was expanded in high-glucose DMEM (Cytiva, Marlborough, MA, USA, cat. SH30243.02) containing 10% fetal bovine serum (Cytiva, cat. SV30160.03) and 1% penicillin/streptomycin (Cytiva, cat. SH40003.01). Cultures were maintained at 37 °C in a humidified 5% CO_2_ incubator. Once cells reached ~90% confluence, they were washed twice with PBS (0.0067 M phosphate, Cytiva, cat. SH302560.1) and detached with 0.25% trypsin-EDTA (Corning, New York, NY, USA, cat. 25-053-CI). A total of 1 × 10^4^ cells were dispensed into each well of 96-well microplates (NEST Biotechnology, Wuxi, China, cat. 701001) and allowed to attach overnight. The following day, cells were treated with MSN-NH_2_ or MSN-CAF at concentrations of 5, 10, 25, 50, 75, and 100 µg/mL (three independent experiments, each performed in triplicate). After 24 h or 48 h of exposure, metabolic activity was quantified with the MTT reduction assay (Sigma-Aldrich, cat. M5655, St. Louis, MO, USA). MTT was added to a final concentration of 0.5 mg/mL, and plates were incubated for 90 min at 37 °C. Formazan crystals were dissolved in dimethyl sulfoxide (DMSO) (Sigma-Aldrich, cat. D8418, St. Louis, MO, USA), and absorbance was recorded at 570 nm on a Synergy H1 reader (BioTek, Winooski, VT, USA) after 2 min of orbital shaking.

### 2.9. Statistical Analysis

Results are expressed as the mean ± SD of three independent experiments, unless otherwise specified by USP guidelines. Statistical significance was determined at *p* < 0.05 using one-way or two-way ANOVA, as appropriate, followed by Tukey’s post hoc test, performed with GraphPad Prism version 8.0.1 (San Diego, CA, USA).

## 3. Results and Discussion

### 3.1. Characterization of Antioxidant Nanomaterial

**Figure 1:**
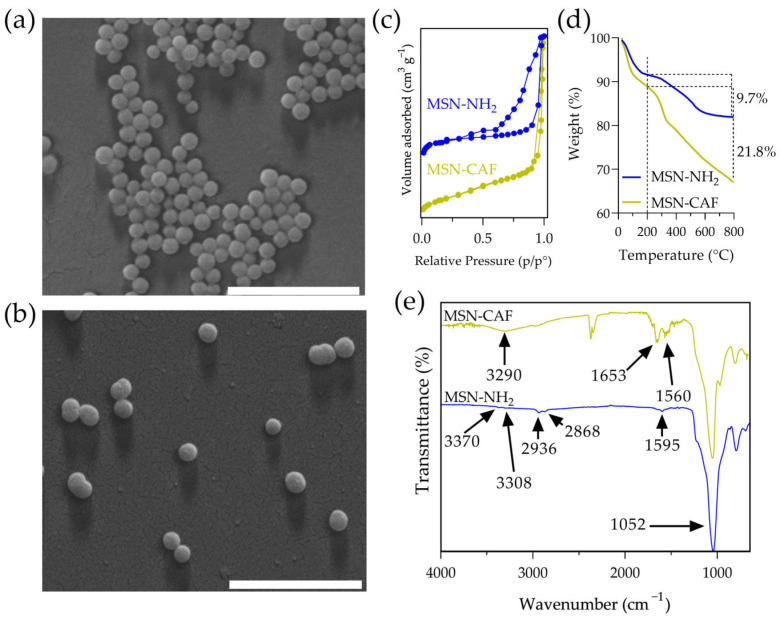
Characterization of nanomaterials. SEM images of (**a**) MSN-NH_2_ and (**b**) MSN-CAF, (**c**) N_2_ adsorption–desorption isotherm of nanomaterials, (**d**) TGA of nanomaterials, and (**e**) FTIR of nanomaterials. Scale bar: 1 µm.

Antioxidant nanomaterials were synthesized via a co-condensation method using TEOS and APTES in a 4:1 ratio, followed by conjugation of caffeic acid to the surface using EDC/NHS chemistry. Physicochemical and textural properties are summarized in Table 4. Monodisperse MSN-NH_2_ samples were obtained with a hydrodynamic diameter of 260 nm and a PdI of 0.15. This was corroborated by SEM images, which revealed spherical particles with an average size of 128 nm (Figure 1a). The zeta potential was +32.5 mV, indicating good colloidal stability attributed to the ionization of amino groups. The N_2_ adsorption–desorption isotherm exhibited a characteristic type IV isotherm indicative of mesoporous materials (Figure 1c). The textural properties of these starting materials included a specific surface area of 542 m^2^/g, a pore diameter of 2.5 nm, and a pore volume of 0.41 cm^3^/g.

Conjugation of caffeic acid, achieved through amide bond formation between the carboxylic acid group of caffeic acid and the amine groups on the silica nanoparticles, yielded ca. 12.1 mg of caffeic acid per 100 mg of nanoparticles (Figure 1d). This value was consistent with previous reports [32]. The resulting nanoantioxidant exhibited a hydrodynamic diameter of 207 nm and a zeta potential of +11.8 mV, indicating partial neutralization of the amino groups and successful conjugation. SEM images revealed no significant changes in nanoparticle morphology and size (Figure 1b). However, the specific surface area and pore volume decreased to 301 m^2^/g and 0.32 cm^3^/g, respectively. On the other hand, FTIR analysis revealed characteristic peaks that confirmed caffeic acid conjugation (Figure 1e). The spectrum of MSN-NH_2_ showed a peak at 1052 cm^−1^, corresponding to siloxane groups, and two weak peaks at 3370 cm^−1^ and 3308 cm^−1^, attributed to primary amine stretching vibrations. Additionally, the spectrum showed peaks at 2936 cm^−1^ and 2868 cm^−1^, attributed to the propyl chain, and a peak at 1595 cm^−1^, characteristic of N-H bending [48]. The intensity of this amine group peak was notably reduced in the nanoantioxidant spectrum, accompanied by the appearance of characteristic bands indicative of amide bond formation at 1653 cm^−1^ and 1560 cm^−1^. In addition, a band centered at 3290 cm^−1^ suggested the presence of aromatic –OH vibrations due to the catechol moiety in caffeic acid. Furthermore, bands attributable to aromatic stretching (C=C) in the range of 1460–1320 cm^−1^ confirmed the presence of caffeic acid.

The antioxidant properties of free caffeic acid (CAF) and the antioxidant nanomaterial (MSN-CAF) were characterized using widely validated methods, including total phenolic content (TPC), DPPH^●^, ABTS^●+^, ORAC, and FRAP assays. The results are presented in Table 5.

The TPC was determined to be 47.5 mg GAE/g for free CAF and 16.0 mg GAE/g for MSN-CAF. In contrast, the TPC for caffeic acid immobilized on the silica surface of MSN-CAF was approximately three times lower. This reduction was expected, as the caffeic acid content was “diluted” within the total mass of the nanomaterial, unlike free CAF, which is a pure polyphenol. The lower TPC in MSN-CAF likely resulted from both the reduced proportion of CAF and its limited availability to react with the Folin-Ciocâlteu reagent.

The DPPH^●^ and ABTS^●+^ assays were quantified using SC_50_ values, where a lower SC_50_ indicates greater antiradical capacity. Free CAF exhibited SC_50_ values of 6.19 μg/mL (DPPH^●^) and 6.02 μg/mL (ABTS^●+^), reflecting its potent antiradical activity. However, when conjugated to nanoparticles in MSN-CAF, the SC_50_ values increased significantly to 86.6 μg/mL (DPPH^●^) and 60.5 μg/mL (ABTS), representing a 10- to 14-fold reduction in antiradical efficiency. These findings suggest that the conjugated form of CAF in MSN-CAF is less effective at scavenging free radicals. Similar trends have been observed by other researchers studying CAF [32,49], gallic acid [50], epigallocatechin-3-gallate [51], and kaempferol [52] incorporated into mesoporous silica nanoparticles (MSNs).

The antiradical activity of caffeic acid is widely attributed to hydrogen atom transfer (HAT) and single electron transfer (SET) mechanisms, with HAT being predominant [19,53,54,55]. This scavenging effect relies on the catechol group in caffeic acid, supported by the stabilizing effects of the benzene ring, the conjugated double bond, and the carboxyl group [56]. However, steric hindrance caused by the nanoparticle limits the accessibility of DPPH^●^ and ABTS^●+^ molecules to CAF, reducing reaction efficiency [55,57]. This restricted diffusion of reactants (DPPH, ABTS, and caffeic acid) is the primary cause of the diminished antioxidant activity in MSN-CAF. Nevertheless, the nanomaterial retained significant radical-scavenging capacity. Conversely, some studies have reported enhanced antiradical capacity when polyphenols are incorporated into nanomaterials [58,59,60], though this is often due to the release of antioxidants from the matrix.

The ORAC and FRAP assays assess the ability of samples to inhibit fluorescein oxidation by reactive oxygen species (ROS) and to reduce the ferric tripyridyltriazine complex to its ferrous form, respectively. Free CAF demonstrated high antioxidant capacity with ORAC = 2083.6 μmol Trolox/g and FRAP = 237.8 μmol Trolox/g. In contrast, MSN-CAF showed reduced values of ORAC = 219.6 μmol Trolox/g and FRAP = 46.8 μmol Trolox/g, reflecting five- and ninefold decreases in antioxidant capacity for FRAP and ORAC, respectively. The consistent reduction in antioxidant capacity across all assays for MSN-CAF can be attributed to three main factors: (1) conjugation effects, such as amide bond formation, which eliminates the carboxyl group’s contribution to antioxidant activity; (2) the proportion of caffeic acid available on the nanoparticle surface relative to that within its pores; (3) limited molecular interactions due to restricted diffusion.

Despite this attenuation, the MSN-CAF antioxidant activity suggests potential for protecting oxidation-sensitive molecules in applications such as biomedicine, pharmaceuticals, cosmetics, or food industries, enhancing product shelf life. Notably, the starting material, MSN-NH_2_, exhibited no significant antioxidant capacity, as previously reported [51,61]. Thus, the observed antioxidant properties of MSN-CAF are directly attributable to the successful conjugation of caffeic acid.

### 3.2. Effect of Nanoparticles on the Rheological Properties of Blended Powders

Tablet formulation requires a minimal set of essential excipients that, together with the active pharmaceutical ingredients (APIs) or bioactive ingredients, ensure adequate powder flowability and compressibility. Generally, direct compression tablet production necessitates the inclusion of a filler, a binder, and a disintegrant; additionally, from an operational perspective, a lubricant is typically required. Based on its established use in pharmaceutical formulations and favorable properties for direct compression [62,63,64], MicroceLac^®^ 100 (a coprocessed filler/binder composed of 75% lactose monohydrate and 25% microcrystalline cellulose), crospovidone (superdisintegrant, 2%), and magnesium stearate (lubricant/glidant, 0.5%) were selected as excipients in addition to the antioxidant nanomaterial. Furthermore, the nonionized nature of crospovidone minimizes the potential for incompatibilities with the nanoparticles.

Powder flowability is a critical factor influencing the manufacturing of tablets produced by direct compression. Accordingly, the excipients and the excipient mixtures with MSN-NH_2_ and MSN-CAF were characterized based on their rheological parameters to ensure adequate flowability for direct compression. Initially, the influence of the antioxidant nanomaterial on the rheological properties of the blended powders was investigated. To this end, the angle of repose, Hausner ratio, and Carr’s index were evaluated for the MSN-CAF nanoparticle powder alone. The angle of repose reflects the degree of interparticle friction or resistance to powder flow. On the other hand, the Hausner ratio and Carr’s index are empirically derived parameters used to evaluate powder flowability based on bulk density measurements [65]. The Hausner ratio serves as a measure of frictional forces between powder particles, while Carr’s index, also known as the compressibility index, quantifies a powder’s tendency to consolidate, derived from the difference between bulk and tapped densities. These parameters highlight the significance of interparticle interactions, which are typically minimal in free-flowing powders, resulting in similar bulk and tapped density values [37].

The MSN-CAF nanoparticle powder displayed an angle of repose of 62°, aligning with a Hausner ratio of 1.51 and a Carr’s index of 34, indicating very poor flow properties (Table 6). A similar pattern was observed for MSN-NH_2_. Bare mesoporous silica nanoparticles (MSNs) generally exhibit hydrophilic nature due to surface silanol (–OH) groups. The starting material in this work, MSN-NH_2_, comprises aminated nanoparticles, which have predominantly aminopropyl moieties on their surface and within pores, alongside residual silanols from TEOS. After conjugation with caffeic acid, partial neutralization of amino groups was observed, likely resulting from amide bond formation. This suggests the coexistence of silanol (–OH), amino (–NH_2_), and caffeic acid moieties on the nanoparticle surface. However, the propyl chain from APTES, the amide bonds formed during conjugation, and the benzene ring of caffeic acid confer a degree of hydrophobicity relative to bare MSNs, leading to slight particle aggregation. Furthermore, residual –NH_2_ groups (due to incomplete functionalization) and catechol groups from caffeic acid may enhance interparticle interactions. These characteristics underscore the necessity of blending MSN-CAF with suitable excipients to facilitate its use in the formulation of oral tablets. Previous studies have similarly reported elevated angles of repose (≥40°) for silica-based materials alone, which is consistent with our results [66,67,68]. In the formulated blends, magnesium stearate and crospovidone were incorporated at a constant low concentration. The ratio of MSN-CAF (or MSN-NH_2_) to MicroceLac^®^ 100 was varied, as detailed in Table 1, yielding four formulations for MSN-CAF (F1–F4) and four formulations for MSN-NH_2_ (N1–N4). Blending the antioxidant nanomaterial with excipients altered the flow properties, as presented in Table 6. Furthermore, a blend of excipients alone (MicroceLac^®^ 100, crospovidone, and magnesium stearate) was evaluated as a control to assess the influence of nanomaterials on flow properties and was designated as B0. This sample (B0) exhibited excellent to good flow properties.

Formulation F4, with 70% MSN-CAF, exhibited flow properties classified as poor to very, very poor. Likewise, formulation F3, containing 50% MSN-CAF, showed a fair angle of repose but very poor Hausner ratio and Carr’s index values. In contrast, formulation F2, with 27.5% MSN-CAF, achieved an angle of repose of 25°, categorized as excellent, with Hausner ratio and Carr’s index values rated as passable. At the lowest MSN-CAF concentration of 10% (F1), the angle of repose was 12°, with a Hausner ratio of 1.16 and a Carr’s index of 14%. These values for F1 indicate excellent flowability, characterized by low cohesiveness and free-flowing behavior, suggesting a system with good consolidation and minimal interparticle friction. This facilitates handling in automated systems such as hoppers, conveyors, or tablet compression equipment. Similarly, formulations N1 to N4 exhibited results that followed the same trend. This behavior underscores that, under our experimental conditions, the antioxidant nanomaterial significantly influenced powder flow properties, with the nanoparticle itself exerting a greater impact than the antioxidant. This can be explained by the fact that caffeic acid, in addition to being conjugated on the surface, is located within the pores, reducing its surface presence, which is in concordance with the antioxidant capacity characterization. Further experiments using a broader range of antioxidants with varying hydrophilic/hydrophobic properties and/or nonporous compact silica nanoparticles could provide deeper insights into these results. Nevertheless, the influence of the antioxidant nanomaterial on powder flow properties was evident and can be attributed to MSN-CAF enhancing interparticle attractive forces, promoting aggregation, and increasing cohesiveness and flow resistance. Formulations F3 and F4, exhibiting poor flowability, high cohesiveness, and limited compaction capacity, may present challenges in industrial-scale material handling and processing. From a technological perspective, only formulations F1 and F2 are suitable for pharmaceutical manufacturing, where powders with passable or better flow properties are preferred for direct compression.

Other studies have reported the influence of colloidal silica on powder flow properties; however, most of them utilized low concentrations [16,69,70]. In the present work, we used antioxidant nanomaterial concentrations ranging from 10% to 70%. Additionally, the minimum concentration (10%) of antioxidant nanomaterials ensured a theoretical antioxidant concentration of approximately 1%, which is within the typical range for antioxidant agents in pharmaceutical formulations [71]. The general flow properties of powders containing antioxidant nanomaterials result from particle geometry, friction, and cohesive forces, which are influenced by the distribution of nanoparticles on powder grains. At higher MSN-CAF concentrations, above approximately 30%, nanoparticle agglomeration reduced the free-flowing nature of the powder, impairing its compactability. These findings are critical because they reveal a threshold for suitable flow properties, establishing guidelines for the future design of oral tablets containing nanoantioxidants.

**Figure 2:**
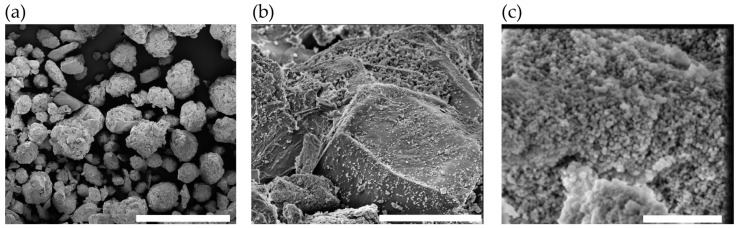
Morphology of the powder of formulation 1. SEM image of (**a**) formulation F1 (scale bar: 400 µm), (**b**) F1, magnification of 13,000× (scale bar: 10 µm), and (**c**) F1, magnification of 100,000× (scale bar: 1 µm).

The morphology of the optimal formulation (F1) was examined using scanning electron microscopy. SEM images revealed particles primarily consisting of spherical, porous microagglomerates (Figure 2a) attributable to the characteristic morphology of MicroceLac^®^ 100 and crospovidone [72,73,74]. The spherical particle shape correlates strongly with the excellent flowability observed. Higher-magnification images (Figure 2b) confirmed the presence of MSN-CAF nanoparticles adsorbed on the particle surfaces. Nevertheless, some areas exhibited a higher density of nanoparticles than others (Figure 2c). If the tablet does not disintegrate properly, this agglomeration of nanoparticles could hinder their free movement during the release process, necessitating evaluation based on critical quality attributes.

### 3.3. Critical Quality Attributes of Tablets

**Figure 3:**
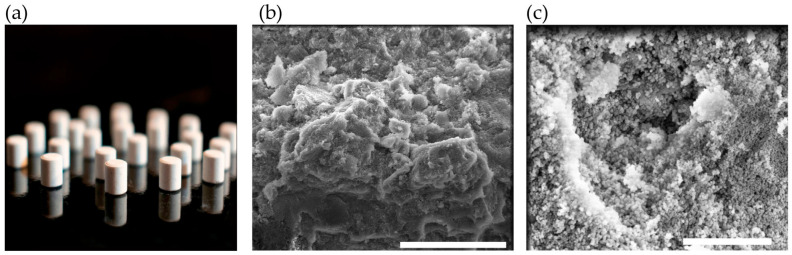
Morphological characteristics of tablets: (**a**) photograph of the tablet; (**b**) SEM image of a tablet cross-section (scale bar: 20 µm); (**c**) SEM image of a tablet cross-section at 50,000× magnification (scale bar: 2 µm).

Based on the results, formulation F1 exhibited the best flow characteristics and was used to manufacture tablets via direct compression. Tablets (124 ± 0.7 mg) were prepared using 5 mm punches (Figure 3a). The diameter and thickness were 4.96 ± 0.02 mm and 5.08 ± 0.04 mm, respectively. Cross-sectional scanning electron microscopy (SEM) images of the tablets (Figure 3b,c) showed that the nanoparticles were completely distributed. These images also confirmed that the morphology of the antioxidant nanomaterial remains intact. Floating tablets consisting of mesoporous silica nanoparticles (MSNs) have been reported [15] exhibiting a similar nanoparticle distribution pattern on the surface of the powder forming the tablet.

Tablet hardness, defined as the ability to withstand external mechanical forces, is primarily governed by intermolecular forces (e.g., hydrogen bonding, hydrophobic interactions, ionic interactions, and van der Waals forces) and intramolecular forces (e.g., covalent, ionic, and metallic bonds). Appropriate hardness ensures that tablets resist frictional forces during handling while maintaining rapid disintegration upon administration. The average hardness was 66 N, consistent with the typical hardness range reported in the literature [75].

Friability testing revealed a friability of 0.03 ± 0.01%. This result demonstrates that all tested tablets met the United States Pharmacopeia (USP) standards (weight loss ≤ 1%), indicating sufficient mechanical strength to withstand stresses such as vibration or abrasion.

The evaluated tablets were formulated for immediate release, lacking coatings or excipients designed to delay disintegration. All tablets exhibited rapid disintegration times of 18 s, meeting USP standards. This performance was attributed to crospovidone [35]. Alshora et al. (2024) reported disintegration times of 10 s for tablets containing furosemide-loaded nanoparticles formulated with 2% crospovidone [76]. No statistically significant differences were observed in tablet hardness, friability, or disintegration across all data sets (*p* > 0.05).

**Figure 4:**
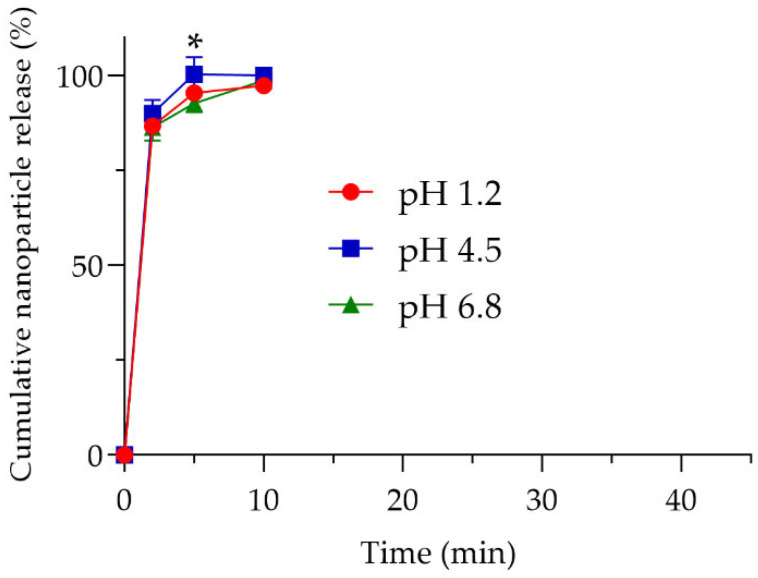
Release profile of the antioxidant nanomaterials. Cumulative nanoparticle release (%) at pH 1.2 (red line), pH 4.5 (blue line), and pH 6.8 (green line). Experiments were performed in triplicate (n = 3). * *p* < 0.05 between pH 6.8 and pH 4.5 at 5 min.

Following disintegration, the tablet releases its contents, enabling MSN-CAF to serve functions beyond acting as an excipient to extend product shelf life, such as an antioxidant agent [1,2] or a drug dissolution rate enhancer [14,77]. Whatever the specific requirement, the nanoparticles must be released from the tablet. To measure nanoparticle release, a drug release study was adapted by weighing nanoparticle pellets from aliquots at predetermined time points. The study was conducted in three media mimicking the gastrointestinal environment (pH 1.2, 4.5, and 6.8). According to the release test, nanoparticles were released rapidly, reaching ~87% release at 2 min, ~96% at 5 min, and ~98% at 10 min (Figure 4), indicating nearly complete release within 15 min. Specifically, at 5 min, samples at pH 6.8 and 4.5 showed statistically significant differences in nanoparticle release (*p* < 0.05).

Nonetheless, the overall results indicated that the nanoparticles were released almost immediately upon contact with simulated gastrointestinal fluids. This rapid release ensures that any subsequent release of a bioactive compound would depend solely on the nanoparticle design and release mechanism rather than being limited by interactions between the nanoparticles and the tablet matrix. The morphology and textural parameters of nanoparticles after the release test were evaluated. The results showed no significant changes (Figures S1 and S2 and Table S1). Taken together, these results demonstrate the successful incorporation of MSN-CAF into oral tablets via direct compression, with suitable critical quality attributes for use as an excipient, drug carrier, antioxidant, or shelf-life extender.

### 3.4. In Vitro Cytotoxicity Studies

**Figure 5:**
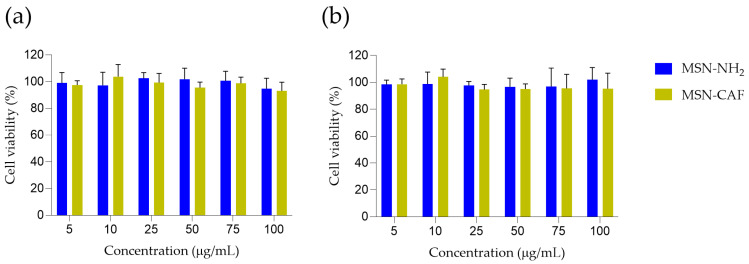
MTT-based analysis of HepG2 cell viability in response to MSN-NH_2_ and MSN-CAF treatments. HepG2 Cells were incubated for (**a**) 24 and (**b**) 48 h with increasing concentrations (5–100 µg/mL) of each formulation. Cell viability was assessed using the MTT assay and expressed as a percentage relative to the untreated control group. Values are presented as mean ± standard deviation from three independent experiments, each performed in triplicate (n = 3). No significant differences were observed between treated and control groups at any concentration or time point (two-way ANOVA, *p* > 0.05).

The excipients in the formulation are well-established, with demonstrated biosafety, and are widely used in pharmaceutical products. Additionally, caffeic acid exhibits hepatoprotective effects [78]. However, the antioxidant nanomaterial described herein requires thorough biocompatibility evaluation. Therefore, we conducted an MTT assay using HepG2 cells, selected for their relevance to the potential oral administration route, to assess the nanomaterial’s biocompatibility. Exposure of HepG2 cells to either MSN-NH_2_ or MSN-CAF for up to 48 h did not compromise cellular metabolic activity (Figure 5).

Mean viabilities at 24 h ranged from 95% to 105% for MSN-NH_2_ and 93% to 104% for MSN-CAF across the concentration range tested. A similar pattern was observed at 48 h, with average values between 97% and 102% for MSN-NH_2_ and 95% to 104% for MSN-CAF. No dose-dependent decline was detected, and statistical analysis revealed no significant differences between treatments groups at any time point (*p* > 0.05). The results demonstrate that HepG2 hepatocytes maintain normal metabolic activity in the presence of both nanoparticle systems at the evaluated doses, suggesting their safety and biocompatibility. These findings are consistent with previous reports on silica-based nanomaterials [79,80,81].

## 4. Conclusions

This study demonstrates the successful incorporation of an antioxidant nanomaterial based on mesoporous silica functionalized with caffeic acid (MSN-CAF) into oral tablets via direct compression, highlighting their potential as a multifunctional platform in pharmaceutical, nutraceutical, and food applications. The reduced antioxidant capacity of MSN-CAF compared with free caffeic acid, observed in DPPH^●^, ABTS^●+^, ORAC, and FRAP assays, suggested limited surface availability due to pore entrapment, yet sufficient activity remained for practical applications. Higher concentrations of the antioxidant nanomaterial in tablet formulations, particularly above approximately 30%, significantly influenced powder flow properties. Specifically, formulation F1 (10% MSN-CAF) exhibited excellent flowability, making it suitable for industrial tablet manufacturing. Furthermore, tablets produced with F1 demonstrated robust critical quality attributes, including rapid disintegration, low friability, and high nanoparticle release. Additionally, an in vitro cytotoxicity assay suggested the biocompatibility of the antioxidant nanomaterial. These results confirm that MSN-CAF can simultaneously function as a pharmaceutical excipient, antioxidant agent, and delivery system. This opens opportunities for its application in active packaging, nutraceuticals, pharmaceuticals, and functional foods, where protection of oxidation-sensitive compounds and controlled release provide added value. Future research could explore other polyphenols, nonporous matrices, or stimuli-responsive systems to optimize bioactive release and expand industrial applications.

## Figures and Tables

**Table 1:** Compositions of the tablet formulations containing antioxidant nanomaterial and MSN-NH_2_.

Excipients	F1 (%)	F2 (%)	F3 (%)	F4 (%)	N1 (%)	N2 (%)	N3 (%)	N4 (%)
MSN-NH_2_	-	-	-	-	10.0	27.5	50.0	70.0
MSN-CAF	10.0	27.5	50.0	70.0	-	-	-	-
MicroceLac^®^ 100	87.5	70	47.5	27.5	87.5	70	47.5	27.5
Crospovidone	2.0	2.0	2.0	2.0	2.0	2.0	2.0	2.0
Magnesium stearate	0.5	0.5	0.5	0.5	0.5	0.5	0.5	0.5
Total	100	100	100	100	100	100	100	100

**Table 2:** Flow properties corresponding to the angle of repose, Hausner ratio, and compressibility index.

Angle of Repose	Hausner Ratio	Compressibility Index	Flow Characteristic
25–30	1.00–1.11	1–10	Excellent
31–35	1.12–1.18	11–15	Good
36–40	1.19–1.25	16–20	Fair
41–45	1.26–1.34	21–25	Passable
46–55	1.35–1.45	26–31	Poor
56–65	1.46–1.59	32–37	Very poor
>66	>1.60	>38	Very, very poor

**Table 3:** Buffer compositions (1 L) for release tests at pH 1.2, 4.5, and 6.8.

Component	pH 1.2	pH 4.5	pH 6.8
Sodium chloride	2.0 g	-	-
Hydrochloric acid	7.0 mL	-	-
Sodium acetate	-	2.99 g	-
Glacial acetic acid	-	1.66 mL	-
Anhydrous dibasic sodium phosphate	-	-	21.72 g
Citric acid monohydrate	-	-	4.94 g
Water	q.s. 1000 mL	q.s. 1000 mL	q.s. 1000 mL

**Table 4:** Physicochemical and textural properties of the synthesized nanomaterials.

Sample	Hydrodynamic Diameter (nm)	Zeta Potential (mV)	Specific Surface Area (m^2^/g)	Pore Diameter (nm)	Pore Volume (cm^3^/g)
MSN-NH_2_	260	+32.5	542	2.5	0.41
MSN-CAF	207	+11.8	301	2.6	0.32

**Table 5:** Antioxidant characterization of nanomaterials. All experiments were performed in triplicate (n = 3).

		Antioxidant Characterization			
Sample	TPC ^a^	DPPH ^b^ SC_50_	ABTS ^b^ SC_50_	ORAC ^c^	FRAP ^c^
CAF	47.50 ± 0.02	6.19 ± 0.01	6.02 ± 0.01	2083.6 ± 3.8	237.8 ± 0.01
MSN-CAF	16.00 ± 0.01	86.60 ± 0.01	60.50 ± 0.01	219.6 ± 4.8	46.80 ± 0.01

^a^ expressed as mg GAE/g MSN-CAF, ^b^ expressed as µg/mL, ^c^ expressed as µmol Trolox/g MSN-CAF.

**Table 6:** Flow properties of MSN-CAF, formulations F1–F4, and formulations N1–N2, determined by angle of repose, Hausner ratio, and Carr’s index (flow characteristics in parentheses). Additionally, B0 represents the blend of excipients (MicroceLac^®^ 100, Crospovidone, and Magnesium stearate).

Sample	Angle of Repose	Hausner Ratio	Carr Index
B0	14 (excellent)	1.13 (good)	11 (good)
MSN-NH_2_	61 (very poor)	1.53 (very poor)	35 (very poor)
MSN-CAF	62 (very poor)	1.51 (very poor)	34 (very poor)
F1	12 (excellent)	1.16 (good)	14 (good)
F2	25 (excellent)	1.29 (passable)	22 (passable)
F3	40 (fair)	1.61 (very poor)	33 (very poor)
F4	46 (poor)	1.58 (very, very poor)	38 (very, very poor)
N1	16 (excellent)	1.14 (good)	12 (good)
N2	27 (excellent)	1.28 (passable)	22 (passable)
N3	37 (fair)	1.53 (very poor)	35 (very poor)
N4	55 (poor)	1.65 (very, very poor)	39 (very, ver poor)

## Data Availability

The manuscript and Supplementary Materials contain the reported data. Additional relevant data can be obtained upon request from the corresponding author.

## Supplementary Materials

The following supporting information can be downloaded at: https://www.mdpi.com/article/10.3390/antiox14070829/s1. Table S1. Textural properties of nanoparticles after release studies at different pH values. Figure S1. SEM images of MSN-CAF after the release study at (a) pH 1.2, (b) pH 4.5, (c) pH 6.8, and (d) pH 6.8 after 5 h. The scale bars in (a–c) represent 5 µm, and in (d) represent 1 µm. Figure S2. N_2_ adsorption/desorption isotherms of MSN-CAF after the release study at (a) pH 1.2, (b) pH 4.5, (c) pH 6.8, and (d) pH 6.8 after 5 h.
