# Supplementary material for: Influence of an Antioxidant Nanomaterial on Oral Tablet Formulation: Flow Properties and Critical Quality Attributes
Source: Antioxidants (Basel). 2025 Jul 5;14(7):829. doi: 10.3390/antiox14070829 (PMC12291767; doi:10.3390/antiox14070829)
Supplement: Supplementary file 1 [file antioxidants-14-00829-s001.zip › antioxidants-3703947-supplementary.pdf]

Supplementary materials

Influence of an antioxidant nanomaterial on oral tablet formulation: flow properties and critical quality attributes

Andrea C. Ortiz[1], Javiera Carrasco-Rojas[2], Sofía Peñaloza[2], Mario J. Simirgiotis[3], Lorena Rubio-Quiroz[4], Diego Ruiz[3], Carlos F. Lagos[4,5], Javier Morales[2,*] and Francisco Arriagada[2,*]

[1] Escuela de Química y Farmacia, Facultad de Ciencias, Universidad San Sebastián, Santiago 7510157, Chile.

[2] Departamento de Ciencias y Tecnología Farmacéutica, Facultad de Ciencias Químicas y Farmacéuticas, Universidad de Chile, Santiago 8380494, Chile.

[3] Instituto de Farmacia, Facultad de Ciencias, Universidad Austral de Chile, Campus Isla teja, Valdivia 5090000, Chile.

[4] Chemical Biology & Drug Discovery Lab, Escuela de Química y Farmacia, Facultad de Ciencias, Universidad San Sebastián, Campus Los Leones, Lota 2465 Providencia 7510157, Santiago, Chile.

[5] Centro Basal Ciencia & Vida, Fundación Ciencia & Vida, Av. del Valle Norte 725, Huechuraba 8580702, Santiago, Chile.

*Corresponding Authors:

Dr. Francisco Arriagada

E-mail address: francisco.arriagada@ciq.uchile.cl

Dr. Javier Morales

E-mail addres: javiermv@ciq.uchile.cl

Table S1. Textural properties of nanoparticles after release studies at different pH values.

Sample	Specific surface area (m^2/g)	Pore diameter (nm)	Pore volume (cm^3/g)
MSN-CAF	301	2.6	0.32
MSN-CAF_pH 1.2	303	2.5	0.31
MSN-CAF_pH 4.5	298	2.4	0.32
MSN-CAF_pH 6.8	300	2.1	0.35
MSN-CAF_pH 6.8_5h	287	2.1	0.31

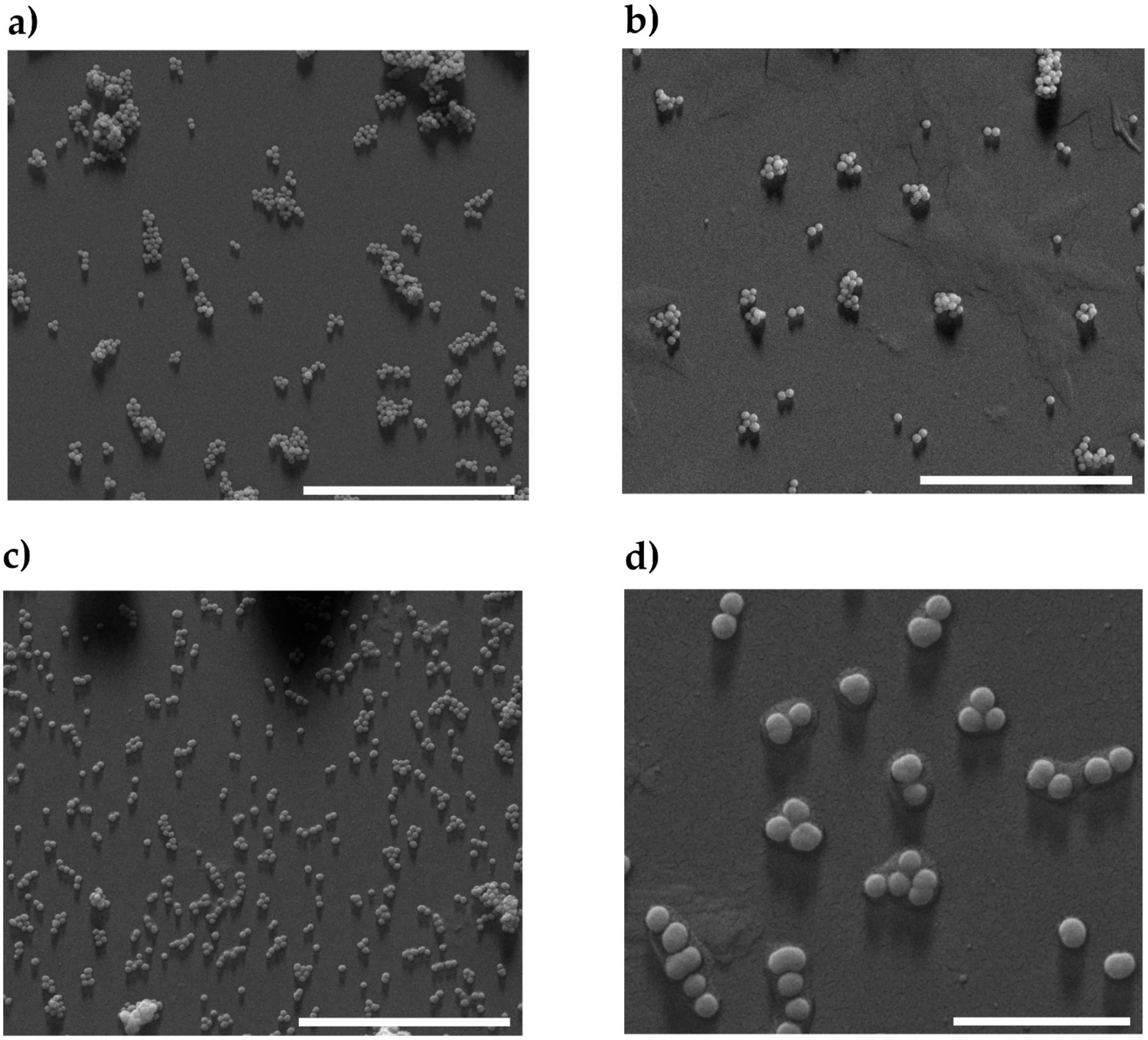

Figure S1. SEM images of MSN-CAF after the release study at a) pH 1.2, b) pH 4.5, c) pH 6.8, and d) pH 6.8 after 5 h. The scale bars in a), b), and c) represent 5 μm, and in d) represent 1 μm.

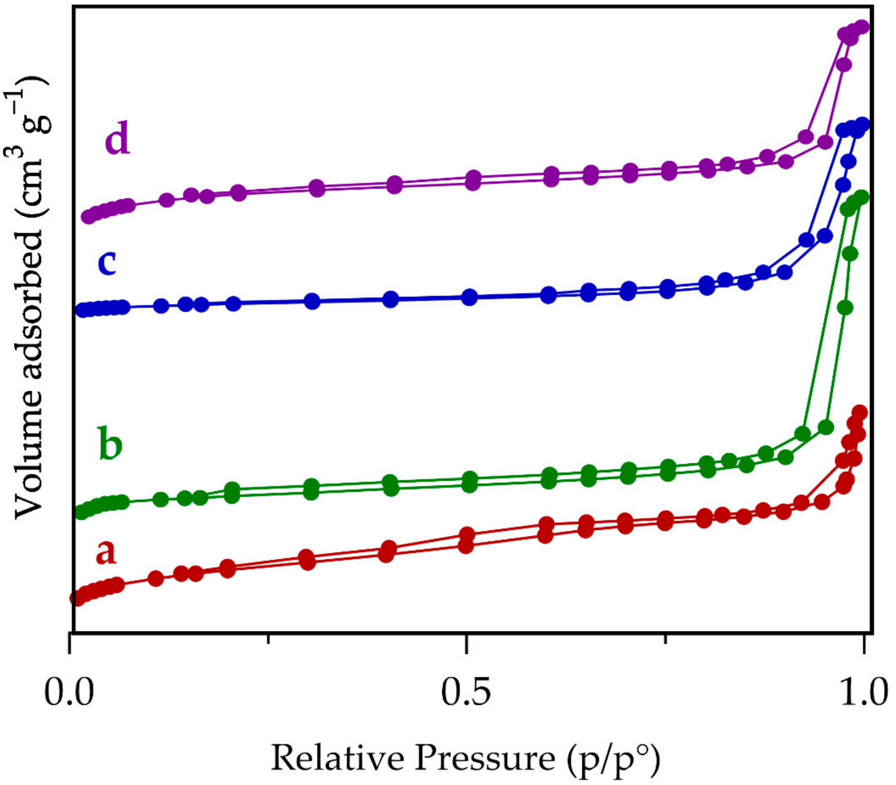

Figure S2. N_2 adsorption/desorption isotherms of MSN-CAF after the release study at a) pH 1.2, b) pH 4.5, c) pH 6.8, and d) pH 6.8 after 5 h.
